# Nonmedical prescription opioid use and illegal drug use: initiation trajectory and related risks among people who use illegal drugs in Vancouver, Canada

**DOI:** 10.1186/s13104-018-3152-9

**Published:** 2018-01-16

**Authors:** Tessa Cheng, Will Small, Ekaterina Nosova, Bob Hogg, Kanna Hayashi, Thomas Kerr, Kora DeBeck

**Affiliations:** 1 Faculty of Health Sciences, Simon Fraser University, Blusson Hall, Room 11300, 8888 University Drive, Burnaby, B.C. V5A 1S6 Canada; 2 British Columbia Centre for Excellence in HIV/AIDS, St. Paul’s Hospital, 608-1081 Burrard Street, Vancouver, B.C. V6Z 1Y6 Canada; 3 Department of Medicine, University of British Columbia, St. Paul’s Hospital, 608-1081 Burrard Street, Vancouver, B.C. V6Z 1Y6 Canada; 4 School of Public Policy, Simon Fraser University, 515 West Hastings Street, Suite 3271, Vancouver, B.C. V6B 5K3 Canada

**Keywords:** Prescription opioid, Addiction, Risk behaviour, Street youth

## Abstract

**Objective:**

We investigated the prevalence of and risk factors associated with initiating nonmedical prescription opioid use (NMPOU) before and after illegal drugs using data from two linked cohort studies of street youth and adults who use illegal drugs in Vancouver, Canada. All participants who attended a study visit between 2013 and 2016 were eligible for the primary analyses.

**Results:**

Among 512 youth and 833 adult participants, the prevalence of NMPOU was extremely high (88% among street youth; 90% among adults), and over one-third of those who reported engaging in NMPOU had initiated NMPOU before illegal drug use (vs. transitioning from illegal drugs to NMPOU). Participants who reported either transitioning to or from NMPOU had higher risk profiles, particularly related to substance use, when compared with those who reported never engaging in NMPOU. Sub-analyses restricted to only those who engaged in NMPOU found few statistically significant differences between those who initiated NMPOU prior to illegal drugs versus those who initiated illegal drugs prior to NMPOU. Findings suggest that among people who use illegal drugs, early NMPOU trajectories do not appear to critically shape future patterns and practices.

**Electronic supplementary material:**

The online version of this article (10.1186/s13104-018-3152-9) contains supplementary material, which is available to authorized users.

## Introduction

As nonmedical prescription opioid use (NMPOU) continues to rise across North America, researchers have identified an alarming trend of individuals initiating NMPOU and then later transitioning to using illegal drugs, such as heroin, cocaine, crack, and crystal methamphetamine [1–5]. Among a sample of people who use heroin in the United States, researchers found that the prevalence of engaging in NMPOU before transitioning to heroin use increased from 64% in 2002–2004 to 83% in 2008–2010 [6]; the prevalence of this particular trajectory was 40% among young heroin injectors in San Diego [5].

Previous research has found key differences in employment and education outcomes between those who engage in illegal drug use and those who engage in NMPOU [7–9]; however, fewer studies have compared transitions to and from NMPOU with those who only use illegal drugs, as well as within-group differences among those who engage in NMPOU. Given these gaps in knowledge, the present study investigates the prevalence of, and risk factors associated with, transitioning from NMPOU to illegal drugs vs. transitioning from illegal drugs to NMPOU use among a sample of street youth and adults who use illegal drugs in Vancouver, Canada.

## Main text

### Methods

Data for this cross-sectional research are drawn from two open prospective cohort studies of youth and adults who use illegal drugs with harmonized procedures and survey instruments: the At-Risk Youth Study (ARYS) and the Vancouver Injection Drug Users Study (VIDUS). Recruitment for both cohorts uses extensive snowball sampling, self-referral, and street outreach. The eligibility criteria for participating in ARYS includes: being between the ages of 14 and 26; use of an illegal drug other than, or in addition to, cannabis in the past month; and “street-involvement”, defined as being recently homeless or having used services designated for street youth [10–13]. The VIDUS cohort includes adults (≥18 years of age) who are HIV-negative and who injected drugs at least once in the previous month. All participants must provide written informed consent to participate. At baseline and every 6 months thereafter, participants in both cohorts complete a harmonized interviewer-administered questionnaire and receive a stipend ($30 CDN) for their time. The ARYS and VIDUS studies receive ethical approval from the University of British Columbia/Providence Health Care Research Ethics Board.

All ARYS and VIDUS participants were eligible for the primary statistical analyses, which were two analyses investigating risk factors associated with (i) transitioning from NMPOU to illegal drug use, and (ii) transitioning from illegal drug use to NMPOU; the comparison group for both analyses were participants who reported never engaging in NMPOU. A report of NMPOU was defined as ever engaging in injection or non-injection NMPOU (yes vs. no) between 2013 and 2016. Transitions to and from NMPOU were categorized based on responses to the following question: “Did you use prescription opioids when they were not prescribed for you or that you took only for the experience or feeling they caused before you had ever used any of the following hard illegal drugs: heroin, cocaine, crack, or crystal methamphetamine?” (yes, non-medical use of POs came before other hard drug use vs. no, non-medical use of POs came after other hard drug use).

The following socio-demographic, early-life, and mental health variables of interest were included: age per year older; male gender (male vs. female); Caucasian ancestry (white vs. non-white); ever experienced homelessness, defined as having no fixed address, sleeping on the street, couch surfing, or staying in a shelter or hostel (yes vs. no); high school incompletion (yes vs. no); a baseline score of 13 or higher on the Childhood Trauma Questionnaire (CTQ), which indicates moderate to severe abuse due to physical abuse, sexual abuse, emotional abuse, physical neglect, and emotional neglect (yes vs. no); and a baseline score of 22 or higher on the Center for Epidemiological Studies Depression Scale (CES-D), which indicates a relatively higher level of depressive symptoms among vulnerable individuals [14] (yes vs. no). Multiple variables related to substance use patterns are also included: daily injection or non-injection heroin use (yes vs. no); daily injection or non-injection of stimulant drugs, including daily use of either crack cocaine, cocaine, or crystal methamphetamine use (yes vs. no); binge drug use, defined as a period of using injection or non-injection drugs more often than usual (yes vs. no); amount of money spent on drugs per day (< median vs. ≥ median); ever experiencing a non-fatal drug overdose (yes vs. no); and ever accessing methadone treatment, which was the most widely available form of opioid agonist treatment in this setting during the study period [15] (yes vs. no). This analysis also includes a range of socio-structural risk factors hypothesized to be associated with this transition pattern: emergency room visit (yes vs. no); experience of violence (yes vs. no); ever been incarcerated (yes vs. no); regular employment, defined as having a regular job, temporary work, or being self-employed (yes vs. no); drug dealing, defined as selling drugs as a source of income (yes vs. no); and ever engaging in sex work, defined as exchanging sex for money, drugs, gifts, food, clothes, shelter or favours (yes vs. no). All variables refer to activities, behaviours, and experiences in the previous 6 months unless otherwise indicated.

Questions related to NMPOU were added to the ARYS and VIDUS survey instrument in June 2013. For participants reporting NMPOU, data for the outcome were drawn from the first study visit where participants reported ever engaging in NMPOU; data for the independent variables were drawn from participants’ baseline study visit. For participants who did not report engaging in NMPOU between 2013 and 2016, data for the outcome and independent variables were also drawn from participants’ baseline study visit.

To assess factors associated with transitions to and from NMPOU (vs. never engaging in NMPOU), bivariate logistic regression analyses were conducted for ARYS and VIDUS participants separately. For variables significant at *p* < 0.10 in the bivariate analyses, a full multivariate model was constructed. The model with the best overall fit was selected using the Akaike Information Criterion (AIC). All statistical analyses were performed using R version 3.2.4 [16]. All *p*-values are two sided.

Sub-analyses were conducted using a restricted sample of only those who reported engaging in NMPOU. The outcome of these analyses was transitioning from NMPOU to illegal drug use (vs. transitioning from illegal drugs to NMPOU), and these analyses used the same independent variables and statistical approach as the primary analysis.

### Results

A total 512 ARYS and 833 VIDUS participants were eligible for the primary analyses. A high proportion of these cohort participants reported ever engaging in NMPOU during a study visit between 2013 and 2016 (ARYS: n = 452, 88%; VIDUS: n = 750, 90%). Among 512 ARYS participants, 334 (65%) were male, 314 (61%) were of Caucasian ethnicity, and the median age was 24 years (Inter-Quartile Range [IQR] 22–27). The majority of VIDUS participants were male (n = 530, 64%) and Caucasian (n = 487, 59%); the median age was 47 years (IQR 38–54). Within each cohort, 160 (31%) ARYS participants (total n = 512) and 276 (33%) VIDUS participants (total n = 833) reported transitioning from NMPOU to illegal drugs. The descriptive characteristics of ARYS and VIDUS participants are displayed in Table 1, and the bivariate analyses investigating PO-related substance use trajectories are shown in Table 2. The results from the multivariate analyses are displayed in Table 3.

**Table 1:** ARYS and VIDUS participant characteristics stratified by trajectory of nonmedical prescription opioid use (NMPOU) (n = 1345)

Characteristic	ARYS (n = 512)			VIDUS (n = 833)		
	Never NMPOU (*%*) (n = 60)	NMPOU first (%) (n = 160)	Illegal drugs first (%) (n = 292)	Never NMPOU (*%*) (n = 83)	NMPOU first (%) (n = 276)	Illegal drugs first (%) (n = 474)
Age per year older [M (IQR)]	26 (22–28)	24 (21–26)	24 (22–27)	49 (42–56)	48 (38–54)	47 (37–54)
Male gender^a^	37 (61.7)	115 (71.9)	182 (62.3)	51 (61.4)	169 (61.2)	310 (65.4)
Caucasian ancestry^a^	35 (58.3)	97 (60.6)	182 (62.3)	37 (44.6)	158 (57.2)	292 (61.6)
Homeless^a^	55 (91.7)	153 (95.6)	275 (94.2)	72 (86.7)	260 (94.2)	444 (93.7)
High school incompletion^a^	27 (45.0)	56 (35.0)	100 (34.2)	39 (47.0)	140 (50.7)	231 (48.7)
Daily heroin use^a,b,c^	5 (8.3)	45 (28.1)	89 (30.5)	10 (12.0)	77 (27.9)	125 (26.4)
Daily stimulant use^a,b,c,d^	22 (36.7)	43 (26.9)	102 (34.9)	21 (25.3)	82 (29.7)	139 (29.3)
Binge drug use^a,b,c^	21 (35.0)	90 (56.3)	181 (62.0)	22 (26.5)	101 (36.6)	175 (36.9)
$ spent on drugs/day^b,e^	22 (36.7)	64 (40.0)	114 (39.0)	22 (26.5)	108 (39.1)	187 (39.5)
Non-fatal overdose^a,c^	27 (45.0)	90 (56.3)	169 (57.9)	34 (41.0)	177 (64.1)	325 (68.6)
Methadone treatment^a^	7 (11.7)	42 (26.3)	87 (29.8)	39 (47.0)	218 (79.0)	374 (78.9)
Emergency room visit^a,b^	23 (38.3)	58 (36.3)	131 (44.9)	16 (19.3)	82 (29.7)	151 (31.9)
Depression symptoms^a^	26 (43.3)	87 (54.4)	160 (54.8)	40 (48.2)	155 (56.2)	241 (50.8)
Childhood trauma^a^	35 (58.3)	97 (60.6)	195 (66.8)	48 (57.8)	183 (66.3)	313 (66.0)
Experience violence^a,b^	12 (20.0)	62 (38.8)	116 (39.7)	9 (10.8)	48 (17.4)	73 (15.4)
Incarceration^a^	35 (58.3)	96 (60.0)	195 (66.8)	63 (75.9)	246 (89.1)	426 (89.9)
Regular employment^a,b^	27 (45.0)	74 (46.3)	133 (45.5)	24 (28.9)	71 (25.7)	133 (28.1)
Drug dealing^a,b^	10 (16.7)	55 (34.4)	95 (32.5)	6 (7.2)	73 (26.4)	110 (23.2)
Sex work^a^	18 (30.0)	41 (25.6)	85 (29.1)	57 (68.7)	169 (61.2)	272 (57.4)

^a^Comparison is yes vs. no

^b^Refers to activities, behaviours, and experiences in the last 6 months

^c^Includes injection and non-injection drug use

^d^Includes crack cocaine, cocaine, or crystal methamphetamine use

^e^Comparison is <median vs. ≥ median

**Table 2:** Bivariate analyses investigating nonmedical prescription opioid use (NMPOU) before or after illegal drug use (n = 1345)

Characteristic	ARYS (n = 512)				VIDUS (n = 833)			
	NMPOU first (vs. never NMPOU)		Illicit drugs first (vs. never NMPOU)		NMPOU first (vs. never NMPOU)		Illicit drugs first (vs. never NMPOU)	
	Odds ratio (95% CI)	*p*-value	Odds ratio (95% CI)	*p*-value	Odds ratio (95% CI)	*p*-value	Odds ratio (95% CI)	*p*-value
Age per year older	0.91 (0.84–0.99)	0.033	0.94 (0.87–1.02)	0.122	0.98 (0.95–1.00)	0.080	0.98 (0.96–1.00)	0.083
Male gender^a^	1.59 (0.85–2.96)	0.146	1.03 (0.57–1.81)	0.923	0.99 (0.59–1.63)	0.972	1.19 (0.73–1.91)	0.487
Caucasian ancestry^a^	1.06 (0.57–1.93)	0.861	1.13 (0.64–2.00)	0.665	1.66 (1.02–2.74)	0.043	1.99 (1.25–3.21)	0.004
Homeless^a^	1.99 (0.57–6.48)	0.257	1.47 (0.47–3.90)	0.467	2.48 (1.08–5.54)	0.028	2.26 (1.04–4.59)	0.029
High school incompletion^a^	0.60 (0.33–1.12)	0.107	0.59 (0.33–1.05)	0.070	1.09 (0.66–1.80)	0.742	1.03 (0.64–1.66)	0.903
Daily heroin use^a,b,c^	4.30 (1.76–12.95)	0.003	4.82 (2.04–14.19)	0.001	2.82 (1.44–6.08)	0.004	2.61 (1.37–5.53)	0.006
Daily stimulant use^a,b,c,d^	0.63 (0.34–1.20)	0.158	0.93 (0.52–1.67)	0.798	1.25 (0.72–2.22)	0.437	1.23 (0.73–2.13)	0.455
Binge drug use^a,b,c^	2.39 (1.30–4.48)	0.006	3.06 (1.73–5.54)	0.000	1.60 (0.94–2.81)	0.091	1.63 (0.98–2.80)	0.067
$ spent on drugs/day^b,e^	1.18 (0.64–2.21)	0.594	1.13 (0.64–2.05)	0.669	1.77 (1.04–3.10)	0.041	1.80 (1.08–3.10)	0.027
Non-fatal overdose^a,c^	1.57 (0.87–2.87)	0.138	1.68 (0.96–2.95)	0.069	2.58 (1.57–4.28)	< 0.001	3.14 (1.96–5.11)	< 0.001
Methadone treatment^a^	2.69 (1.20–6.91)	0.024	3.21 (1.49–8.00)	0.006	4.24 (2.53–7.16)	< 0.001	4.22 (2.60–6.87)	< 0.001
Emergency room visit^a,b^	0.91 (0.50–1.70)	0.775	1.31 (0.75–2.34)	0.354	1.77 (0.99–3.33)	0.064	1.96 (1.12–3.60)	0.023
Depression symptoms^a^	1.61 (0.85–3.07)	0.145	1.55 (0.85–2.83)	0.148	1.35 (0.79–2.29)	0.271	1.15 (0.69–1.90)	0.593
Childhood trauma^a^	1.08 (0.53–2.15)	0.825	1.17 (0.60–2.21)	0.630	1.58 (0.94–2.66)	0.083	1.61 (0.98–2.62)	0.059
Experience violence^a,b^	2.53 (1.28–5.34)	0.010	2.63 (1.38–5.38)	0.005	1.73 (0.85–3.92)	0.156	1.50 (0.75–3.33)	0.282
Incarceration^a^	1.07 (0.58–1.95)	0.823	1.44 (0.81–2.53)	0.212	2.60 (1.37–4.87)	0.003	2.82 (1.54–5.00)	0.001
Regular employment^a,b^	1.05 (0.58–1.92)	0.868	1.02 (0.59–1.80)	0.938	0.85 (0.50–1.49)	0.564	0.96 (0.58–1.63)	0.873
Drug dealing^a,b^	2.62 (1.28–5.84)	0.012	2.41 (1.22–5.23)	0.017	4.61 (2.08–12.27)	0.001	3.88 (1.78–10.19)	0.002
Sex work^a^	0.80 (0.42–1.57)	0.515	0.96 (0.53–1.79)	0.890	0.72 (0.42–1.21)	0.219	0.61 (0.37–1.00)	0.055

^a^Comparison is yes vs. no

^b^Refers to activities, behaviours, and experiences in the last 6 months

^c^Includes injection and non-injection drug use

^d^Includes crack cocaine, cocaine, or crystal methamphetamine use

^e^Comparison is <median vs. ≥ median

**Table 3:** Multivariate analyses investigating nonmedical prescription opioid use (NMPOU) before or after illegal drug use (n = 1345)

Characteristic	ARYS (n = 512)				VIDUS (n = 833)			
	NMPOU first (vs. never NMPOU)		Illicit drugs first (vs. never NMPOU)		NMPOU first (vs. never NMPOU)		Illicit drugs first (vs. never NMPOU)	
	Adjusted odds ratio (95% CI)	*p*-value	Adjusted odds ratio (95% CI)	*p*-value	Adjusted odds ratio (95% CI)	*p*-value	Adjusted odds ratio (95% CI)	*p*-value
Age per year older							0.98 (0.95–1.01)	0.128
Male gender^a^								
Caucasian ancestry^a^							2.39 (1.38–4.19)	0.002
Homeless^a^								
High school incompletion^a^								
Daily heroin use^a,b,c^	3.76 (1.33–13.52)	0.022	3.68 (1.37–12.79)	0.019				
Daily stimulant use^a,b,c,d^								
Binge drug use^a,b,c^	2.05 (1.08–3.95)	0.030	2.07 (1.12–3.90)	0.022				
$ spent on drugs/day^b,e^								
Non-fatal overdose^a,c^					1.81 (1.03–3.19)	0.038	2.43 (1.42–4.19)	0.001
Methadone treatment^a^	2.22 (0.86–6.51)	0.118	3.13 (1.32–8.68)	0.016	4.43 (2.50–7.96)	< 0.001	3.63 (2.12–6.24)	< 0.001
Emergency room visit^a,b^								
Depression symptoms^a^								
Childhood trauma^a^					1.96 (1.10–3.53)	0.023	1.85 (1.05–3.24)	0.033
Experience violence^a,b^	2.42 (1.18–5.28)	0.020	2.74 (1.36–5.93)	0.007				
Incarceration^a^					1.92 (0.93–3.90)	0.072	2.26 (1.11–4.50)	0.021
Regular employment^a,b^								
Drug dealing^a,b^			1.85 (0.87–4.32)	0.127	4.06 (1.72–11.31)	0.003	2.68 (1.17–7.29)	0.032
Sex work^a^								

^a^Comparison is yes vs. no

^b^Refers to activities, behaviours, and experiences in the last 6 months

^c^Includes injection and non-injection drug use

^d^Includes crack cocaine, cocaine, or crystal methamphetamine use

^e^Comparison is <median vs. ≥ median

A total of 452 ARYS and 750 VIDUS participants reported ever engaging in NMPOU and were eligible for inclusion in the sub-analyses. The full results from the sub-analyses investigating transitions to and from NMPOU can be found in Additional files 1: Table S1 and 2: Table S2 attached to this article. In brief, the bivariate VIDUS analysis revealed no significant risk factors associated with transitioning from NMPOU to illegal drug use (*p* > 0.10); no multivariate analysis was performed. A multivariate analysis was performed for the ARYS participants with male gender, daily illicit stimulant use, and emergency room use eligible for the final model (*p* < 0.10); only male gender was significantly associated with transitioning from NMPOU to illegal drugs in the final multivariate model (Adjusted Odds Ratio [AOR] = 1.57, 95% Confidence Interval 1.04–2.41).

### Discussion

Among our sample of people who use illegal drugs in Vancouver, BC, the prevalence of NMPOU was extremely high (88% among street youth and 90% among adults), and over one-third of those who reported engaging in NMPOU had initiated NMPOU before illegal drug use (vs. transitioning from illegal drugs to NMPOU). Participants who reported either transitioning from NMPOU to illegal drugs or from illegal drugs to NMPOU shared many risk factors when compared with those who reported never engaging in NMPOU. Regardless of their transition trajectory, youth who engaged in NMPOU were significantly more likely to engage in daily heroin use, binge drug use, and experience violence than those who never engaged in NMPOU. Adults who engaged in NMPOU were significantly more likely to report overdose, accessing methadone treatment, a higher score on the Childhood Trauma Questionnaire, and drug dealing regardless of transition trajectory. With the exception of our finding that males in the youth cohort were more likely to transition from NMPOU to illegal drugs (vs. transition from illegal drugs to NMPOU), overall our results indicate that the transition patterns between NMPOU and illegal drugs were not meaningfully different with respect to socio-demographic, early life risk factors, substance use, income generation, or other socio-structural risk factors.

## Limitations

This study did not include a sample based on random recruitment methods, although extensive street-based outreach efforts were undertaken to achieve a diverse sample. In addition, the survey responses in this study were subject to recall and socially desirable response biases; previous research, however, has found that self-reports of drug use and related behaviours are valid [17, 18].

## Additional files

**Additional file 1: Table S1.** Bivariate analyses of participants reporting nonmedical prescription opioid use prior to illegal drugs (n = 1202).

**Additional file 2: Table S2.** Multivariate analysis of ARYS participants reporting nonmedical prescription opioid use prior to illegal drugs (n = 452).

## Abbreviations

AOR: adjusted odds ratio
ARYS: At-Risk Youth Study
CI: confidence interval
NMPOU: nonmedical prescription opioid use
VIDUS: Vancouver Injection Drug Users Study
