# Supplementary material for: Nonmedical prescription opioid use and illegal drug use: initiation trajectory and related risks among people who use illegal drugs in Vancouver, Canada
Source: BMC Res Notes. 2018 Jan 16;11:35. doi: 10.1186/s13104-018-3152-9 (PMC5771131; doi:10.1186/s13104-018-3152-9)
Supplement: Supplementary file 1 — Additional file 1: Table S1. Bivariate analyses of participants reporting nonmedical prescription opioid use prior to illegal drugs (n = 1202). [file 13104_2018_3152_MOESM1_ESM.docx]

**Additional File 1**

| **Table S1. Bivariate analyses of participants reporting nonmedical prescription opioid use prior to illegal drugs (n=1,202).** | | | | | |
| --- | --- | --- | --- | --- | --- |
|  | **ARYS (n=452)** | |  | **VIDUS (n=750)** | |
| Characteristic | **Odds Ratio**  **(95% CI**) | ***p* *-* value** |  | **Odds Ratio**  **(95% CI**) | ***p -* value** |
| Age per year older | 0.97 (0.92 - 1.02) | 0.268 |  | 1.00 (0.99 - 1.01) | 0.9214 |
| Male gender^a^ | 1.54 (1.02 - 2.36) | 0.042 |  | 0.84 (0.61 - 1.14) | 0.2519 |
| Caucasian ancestry^a^ | 0.93 (0.63 - 1.39) | 0.722 |  | 0.83 (0.62 - 1.13) | 0.2404 |
| Homeless^a^ | 1.35 (0.57 - 3.56) | 0.513 |  | 1.10 (0.60 - 2.10) | 0.7697 |
| High school incompletion^a^ | 1.03 (0.68 - 1.54) | 0.895 |  | 1.06 (0.78 - 1.43) | 0.7216 |
| Daily heroin use^a,b,c^ | 0.89 (0.58 - 1.36) | 0.600 |  | 1.08 (0.77 - 1.50) | 0.6494 |
| Daily stimulant use^a,b,c,d^ | 0.68 (0.45 - 1.04) | 0.080 |  | 1.02 (0.73 - 1.41) | 0.9111 |
| Binge drug use^a,b,c^ | 0.78 (0.53 - 1.16) | 0.217 |  | 0.98 (0.72 - 1.34) | 0.9120 |
| $ spent on drugs/day^b,e^ | 1.04 (0.70 - 1.55) | 0.837 |  | 0.98 (0.72 - 1.33) | 0.8917 |
| Non-fatal overdose^a,c^ | 0.94 (0.63 - 1.38) | 0.738 |  | 0.82 (0.60 - 1.12) | 0.2134 |
| Methadone treatment^a^ | 0.84 (0.54 - 1.29) | 0.425 |  | 1.00 (0.70 - 1.45) | 0.9787 |
| Emergency room visit^a,b^ | 0.70 (0.47 - 1.04) | 0.076 |  | 0.90 (0.65 - 1.25) | 0.5402 |
| Depression symptoms^a^ | 1.04 (0.68 - 1.58) | 0.865 |  | 1.17 (0.85 - 1.63) | 0.3315 |
| Childhood trauma^a^ | 0.92 (0.59 - 1.46) | 0.725 |  | 0.99 (0.71 - 1.38) | 0.9396 |
| Experience violence^a,b^ | 0.96 (0.65 - 1.43) | 0.853 |  | 1.16 (0.77 - 1.72) | 0.4750 |
| Incarceration^a^ | 0.75 (0.50 - 1.11) | 0.150 |  | 0.92 (0.57 - 1.51) | 0.7479 |
| Regular employment^a,b^ | 1.03 (0.70 - 1.51) | 0.886 |  | 0.89 (0.63 - 1.24) | 0.4885 |
| Drug dealing^a,b^ | 1.09 (0.72 - 1.63) | 0.691 |  | 1.19 (0.84 - 1.67) | 0.3190 |
| Sex work^a^ | 0.84 (0.54 - 1.29) | 0.430 |  | 1.17 (0.87 - 1.59) | 0.3020 |
| a. Comparison is yes vs. no.  b. Refers to activities, behaviours, and experiences in the last six months.  c. Includes injection and non-injection drug use.  d. Includes crack cocaine, cocaine, or crystal methamphetamine use.  e. Comparison is <median vs. ≥ median. | | | | | |
