# Supplementary material for: Nonmedical prescription opioid use and illegal drug use: initiation trajectory and related risks among people who use illegal drugs in Vancouver, Canada
Source: BMC Res Notes. 2018 Jan 16;11:35. doi: 10.1186/s13104-018-3152-9 (PMC5771131; doi:10.1186/s13104-018-3152-9)
Supplement: Supplementary file 2 — Additional file 2: Table S2. Multivariate analysis of ARYS participants reporting nonmedical prescription opioid use prior to illegal drugs (n = 452). [file 13104_2018_3152_MOESM2_ESM.docx]

**Additional File 2**

| **Table S2. Multivariate analysis of ARYS participants reporting nonmedical prescription opioid use prior to illegal drugs (n=452).** | | |
| --- | --- | --- |
| Characteristic | **Adjusted Odds Ratio**  **(95% CI**) | ***p* *-* value** |
| Male gender^a^ | 1.57 (1.04 – 2.41) | 0.036 |
| Daily stimulant use^a,b,c,d^ | 0.71 (0.46 - 1.09) | 0.119 |
| Emergency room visit^a,b^ | 0.70 (0.47 – 1.05) | 0.087 |
| a. Comparison is yes vs. no.  b. Refers to activities, behaviours, and experiences in the last six months.  c. Includes injection and non-injection drug use.  d. Includes crack cocaine, cocaine, or crystal methamphetamine use. | | |
